# Challenges experienced by mothers caring for children with cerebral palsy in Zambia

**DOI:** 10.4102/sajp.v71i1.274

**Published:** 2015-11-10

**Authors:** Carol Singogo, Margaret Mweshi, Anthea Rhoda

**Affiliations:** 1 Department of Physiotherapy, University of the Western Cape, South Africa; 2 Department of Physiotherapy, University of Zambia, Zambia

## Abstract

**Background:**

Mothers caring for children with disability experience a number of challenges.

**Aim:**

The aim of the study was to explore the challenges that mothers who cared for children with cerebral palsy (CP) living in Zambia experienced.

**Methods:**

During a qualitative study the experiences of 16 conveniently sampled mothers of children with CP, from the Ndola district in Zambia, were explored by means of interviews. The responses were thematically analysed. All the necessary ethical considerations were upheld.

**Results:**

Mothers experienced social isolation and marital problems, as well as negative attitudes from family, friends, community members and health care professionals. The physical environment created access challenges because of a lack of sidewalks, ramps, functioning lifts and small indoor spaces.

**Conclusion:**

Mothers of children with CP feel socially isolated owing to a lack of support from family, community members, and health care providers. This social isolation was exacerbated by attitudes of others towards the mothers; it was felt that mothers were responsible for their children's condition. Mothers also experienced marital problems as a result of having a child with CP.

## Introduction

Cerebral palsy (CP) is the most common cause of neurological impairment in children, and can be associated with lifelong disability (Brannen & Heflinger 2006; Pakula, Van Naarden Braun & Yeargin-Allsopp 2009). Sensory, motor, speech and other cognitive impairments are also experienced by children with CP (Pakula *et al*. 2009). Because of the functional limitations experienced, some children with CP are dependent on others for assistance with daily activities, which leads to long-term caregiving that far exceeds the usual needs of typically developing children (Resch *et al*. 2010). Providing the high level of care required by a child with long-term functional limitations can become burdensome, and may affect both the physical and psychological health of the caregiver (Dambi & Jelsma 2014; Raina *et al*. 2005). Caring for a child with a disability affects the role of both parents, but the daily lives of mothers are often more affected, because they are usually the primary caregivers for the child.

Mothers who care for their children with CP experience many challenges. Broadly, the challenges include psychological ones owing to caregiver demands and uncertainties (Glasscoe *et al*. 2007; Sajedi *et al*. 2010) and physical health challenges that emanate from excessive stress and through constantly assisting their children in activities of daily living (Tonga & Duger 2008). In addition the mothers experience socio-economic challenges because many mothers lack employment opportunities (Borst 2010) as well as marital problems (Vijesh & Sukumaran 2007). The challenges could therefore be viewed within a bio-psychosocial model of disability (World Health Organization [WOH] 2001). It is therefore evident that the quality of life of these mothers is negatively affected as a result of caring for their children with CP (Olawale, Deih & Yaadar 2013; Yilmaz *et al*. 2013).

Caring for a child with CP may be overwhelming, resulting in sorrow and grief as the reality of lost hopes and dreams becomes apparent (Huang, Kellet & St John 2010). It has been reported that, in addition to the emotional problems parents have to deal with, they also have to cope with the negative attitudes of friends, relatives and the community at large (Lynch 2007). These experiences have been described by mothers and caregivers of disabled children in developed countries as well as developing countries including Asia and Africa (Dambi & Jelsma 2014; Geere *et al*. 2013; Sandy, Kgole & Mavundla 2013). Communities in Africa often have a negative perception of people with disabilities because they believe that it is a punishment from God (Urimbenshi & Rhoda 2011) and is of a spiritual nature (Wegner & Rhoda 2015).

To address the challenges experienced by mothers caring for children with disabilities, rehabilitation interventions should take place. As these challenges experienced are multidimensional an inter-sectorial approach based on a model such as the Community-based Guidelines Matrix should be considered (WHO 2010). Such interventions are, however, limited in developing countries (Kengne & Anderson 2006) such as Zambia. Cultural beliefs also affect utilisation of rehabilitation services which in turns affects recovery and outcomes of individuals with disabilities (Wegner & Rhoda 2015). No studies relating to the challenges experienced by mothers in Zambia have, however, been published. It has been recommended that studies need to be conducted which should consider factors that are sensitive to the Zambian culture (Mweshi *et al*. 2011). The aim of this study was to explore the challenges experienced by mothers of children with CP in Ndola, a district in the Copper Belt in Zambia.

## Methodology

### Research setting

The study was conducted in Mushili and Twapia townships of Ndola district. The district is one of the ten districts on the Copper Belt that has a population of 37 299 from 6312 households; of these 2.2% are persons with disabilities and amongst these are children with CP (Loeb 2008). Because of the closure of companies in Ndola, there is a high unemployment rate (60%) and people live in abject poverty. Although most of the houses in Ndola town have electricity, more than half of the houses in Mushili and Twapia are old, small, very close to each other and dilapidated. There is no water supply in most households and there is poor sanitation as most people in the areas use pit latrines for their toilets which are very close to their houses (Central Statistics Office 2002). A rehabilitation centre in Mushili and Twapia was accessed as the specific research sites. These centres are managed by trained paramedical personnel such as clinical officers (COs), nurses and health assistants. Mushili rehabilitation centre has three COs and 20 nurses, and the Twapia rehabilitation centre has two COs and 13 nurses. Mushili rehabilitation centre sees about a 100 new CP cases in a year and Mushili only about 60 new cases of CP per year (personal communication with the physiotherapists in the two centres).

### Study design and sample

An exploratory qualitative research design was applied in this study.

The sampling frame for the study comprised all mothers of children with CP who responded to the invitation to participate in the study. The researcher met with a coordinator at the Catholic Diocese of Ndola in Zambia, who wrote introductory letters and contacted the administrators of the two rehabilitation centres. The physiotherapists contacted the mothers and informed them about the study. All in all, 40 mothers responded. Stratification of the sample was used to group participants into strata relevant to the research question. The strata included age (below and above 30 years), employment status (employed or unemployed), marital status (married or single), and the age of the child (below and above 7 years). The names of the mothers who responded were divided into strata, and the researcher then selected 20 participants according to convenience which included at least one from each strata. By the thirteenth interview, no new data emerged from the participants, but the researcher (C.S.) interviewed three more mothers to ensure that theoretical saturation was reached. An extensive review of literature regarding challenges experienced by mothers of children with CP, which assisted probing if needed, and repetition of challenges as perceived by participants, led the researcher to stop the interviews after the sixteenth mother had been interviewed.

### Data collection

Qualitative data were collected through face-to-face semi-structured individual interviews. The researcher (C.S.) used an interview guide which focused broadly on the psychological, physical, socio-economical and environmental challenges experienced by the mothers. Each interview, which was conducted in the preferred language of the participant, lasted between 45 minutes and an hour, and was audio-recorded. After each interview, the audio recorder was played back to each participant in order to confirm or clarify what was said. These recordings were then stored for later analysis.

### Data analysis

An inductive data analysis approach was used to analyse the data relating to the phenomenon, information which was lacking in the current research setting. Data processing began with verbatim transcriptions of the interviews by the main researcher. A professional translator translated the Bemba transcripts into English, and another independent translator read the English transcripts and translated them back into Bemba and compared them to the original Bemba transcripts to ensure accuracy. The main researcher explored the transcripts carefully by reading them several times to obtain a general sense of the information and to reflect on the overall meaning of the participants’ words as well as to understand the contents as described by Creswell (2009). The data were coded by identifying key words in contexts and word repetitions (Patton 1990). The themes were identified by combining codes with similar meanings. Discussions with three colleagues and peers helped to critique and verify the process of coding, categorising and arranging the data into appropriate themes. A limitation of the study was that only the translation, coding and categorisation process was verified and not the transcription process.

## Ethical considerations

Ethical clearance was received from the University of the Western Cape Ethics Committee. In addition, permission was obtained from the Ministry of Health (MOH) in Zambia, the Tropical Diseases Research Centre's Scientific and Ethical Committee (TDRC-SEC) and the Catholic Diocese. Participation was voluntary and participants were able to withdraw at any point in the study. Written informed consent was obtained from the participants, and permission to use an audio recorder was also obtained from them.

## Results

The sample size for this study was *n* = 16 which comprised mothers of children with CP. The mothers’ ages ranged between 18 and 50 years. The ages of the children ranged between 11 months and 17 years with different types of CP. The mothers’ level of education, marital and employment status are indicated in Table 1.

**TABLE 1:** Demographic data for participants and their children with cerebral palsy.

Participant code	Age of participant	Marital status	Level of education	Status of employment	Age of child (years)
M1	42	Married	Tertiary	Employed	7
M2	37	Divorced	Primary	Unemployed	8
M3	33	Married	Tertiary	Self employed	3
M4	37	Married	None	Unemployed	4
M5	21	Single	Secondary	Self employed	2
M6	50	Married	None	Unemployed	17
M7	25	Single	Primary	Unemployed	11 months
M8	35	Divorced	None	Unemployed	13
M9	18	Married	Secondary	Unemployed	2
M10	38	Widow	None	Self employed	6
M11	42	Divorced	Primary	Employed	7
M12	22	Single	Secondary	Unemployed	5
M13	27	Married	None	Unemployed	7
M14	43	Married	None	Unemployed	10
M15	31	Divorced	Secondary	Unemployed	3
M16	39	Married	None	Unemployed	14

The themes that emerged were physical challenges experienced by the mothers, social isolation, perceived causes of CP, physical access challenges, marital problems and challenges with the health care system. Nearly all the mothers were experiencing some form of these problems because they had a child with CP.

### Physical challenges experienced by the mothers

The mothers’ experienced physical problems as a result of looking after their children. These occurred as a result of having to lift and carry their children whilst assisting the children with their functional activities:

‘He is able to sit on his own, but he cannot crawl or walk. He has to be lifted for all activities. He cries a lot during the night and I cannot sleep … I only rest when my mother visits.’ (M4, female, no education, unemployed, 37 years old)

‘The child has stiff muscles all over the body he cannot move. He does not sit or move. Everything is done for him. You have to feed him, wash him, lift him a lot and clean him after he soils his nappies. That is too much for me to handle considering I have other small kids now. I am constantly tired and have these headaches that don’t go away.’ (M16, female, no education, unemployed, 39 years old)

‘He cannot walk, his legs are weak. I lift him for bathing and to the toilet. At least he talks and feeds himself. It affects me in my mind, I can’t think straight and I suffer from memory losses … He is 13 years old, he should be doing things for himself, but I have to do it for him …’ (M8, female, no education, unemployed, 35 years old)

### Social isolation

The mothers expressed experiencing a lack of support from family, friends and community members. This resulted in them being socially isolated which was partly as a result of fear amongst mothers that people would not accept their children or blame them for the condition of the children:

‘I have not had so much contact with my friends and neighbours as I stay indoors with my child most of the time. I am afraid of being laughed at or other people not accepting my child the way she is, it would hurt. I also don’t want anyone to blame me for the condition.’ (M15, female, secondary education, unemployed, 31 years old)

‘My friends laugh at me. He is a very difficult child, if I go with him to church, we spend the whole time outside so I have stopped going to church, not even town. Unless I’m in dire need then I ask my mother to help look after him …’ (M12, female, secondary education, unemployed, 22 years old)

‘My mother remarried after the death of my father. She does not support me at all as she is afraid that her new husband and his relatives might not accept her if they knew I had a disabled child … We are not even allowed to visit her.’ (M7, female, primary education, unemployed, 25 year old)

‘My husband's family does not like me and the fact that I have a child with CP. They have been trying to force my husband to leave me and marry someone else … My friends say all sorts of things and refer to my child as a mentally ill child and that my husband wants to leave me because of that, they are big snakes in the grass.’ (M4, female, no education, unemployed, 37 years old)

### Perceived causes of the cerebral palsy

According to the mothers they were blamed for the child`s condition by husbands, family, friends, and community members: ‘[*T*]hey [*her friends*] were telling everyone in the community that the baby is sick because of my promiscuous ways [*She sobs*] …’ (M12, female, secondary education, unemployed, 22 years old), and, ‘I struggle and the fact that my husband and his family accused me of being the cause of the condition it is difficult …’ (M4, female, no education, unemployed, 37 years old).

A 38-year-old widow trying to run her restaurant business was accused by the community of bewitching her child for her own gain: ‘They say I have bewitched my child to enrich myself … I used to take him before and people shunned my restaurant saying I was using my child with CP to pull customers to myself’ (M10, female, no education, employed, 38 years old).

### Physical access challenges

Nearly all the mothers experienced physical access problems owing to the natural geography, architectural features in the built environment and transport challenges. These included narrow or no sidewalks, narrow doorways, an absence of ramps, no or broken lifts and small indoor spaces:

‘When we go to town, it is a problem as most shops do not have ramps for wheelchairs. Hardly any of the lifts on the tall buildings work. That is the same at the big hospitals. The lifts don’t work most of the time.’ (M1, female, tertiary education, employed, 42 years old)

‘We stay in a very small two-roomed house, there is no space in the house for the child to play, a wheel chair cannot even fit in the house.’ (M9, female, secondary education, unemployed, 18 years old)

‘The roads are inaccessible, it is difficult when she gets sick, my house is in a mountainous region with a lot of rocks. I am only renting a room. Taxis cannot get to this area … Because of the rocks, it is not safe for my child to play.’ (M8, female, no education, unemployed, 35 years old)

### Marital problems

The interviews revealed that some of the mothers were in conflict with their spouses because of having a child with CP. Some of them divorced as a result. This was mainly because of a lack of acceptance of the child and embarrassment, especially by the male spouses, as well as influence from relatives, poor spousal support and poor coping mechanisms:

‘He was too embarrassed about the condition of the child and did not want others to know about it and sympathise with us. He wanted us to keep the condition of the child a secret, I could not. This put a strain on our relationship as we were always fighting.’ (M3, female, tertiary education, employed, 33 years old)

‘Since he discovered that the child had CP that is how he disappeared. I have not seen him since, I just hear from other people that he is now married to someone else.’ (M2, female, primary education, unemployed, 37 years old)

‘The father ran away upon discovering the child had CP … His relatives have not accepted the condition of the child because when I ask for financial support, they say ‘if the father is not responsible over the child, don’t expect us to be’. It shows that they have not accepted the child.’ (M10, female, no education, employed, 38 years old)

### Challenges with health care system

Challenges that mothers experienced with the health care system included a lack of provision of assistive devices, attitudes of health care professionals and a lack or provision of transport as is illustrated in the quotations below. One of the mothers found health care professionals unhelpful and disrespectful:

‘If the government cared, we would not be suffering the way we do. I have failed to purchase any of the assistive devices I was advised to buy. I don’t even have enough money to take my child for physiotherapy. Do policies exist?’ (M15, female, secondary education, unemployed, 31 years old)

‘The nurse I found on duty gave me a very bad attitude and said the only way she would help me was if my child had TB. I tried to reason with her, but she would not listen and told me to report her to the district health board if I wasn’t happy with the services. Desperate for help, I reported her to the district board which did not turn out well as she was nearly fired. Equally this did not work well for me as now all the nurses started giving me negative attitudes.’ (M11, female, primary education, employed, 42 years old)

‘In Lusaka, mothers are transported to and from the rehabilitation centres under the sponsorship of CP Africa, if there is no transport, the mothers are reimbursed with their bus fares. Why can’t they extend the same help to us or the government should at least provide transport for us. We struggle a lot where transport is concerned … There is an organisation that feeds children with HIV/AIDS. I wish our children could be included in that program …’ (M11 female, primary education, employed, 42 years old)

## Discussion

The mothers of children with CP from Ndola in Zambia experienced several problems as a result of having a child with CP. These challenges were similar to what had been previously reported in the literature, with the attitudinal challenges relating to cultural belief being mainly reported in studies conducted in Africa.

It was clear from the findings of this study that mothers were experiencing physical challenges. This finding was similar to the findings by Dambi and Jelsma (2014). Mothers caring for children with disabilities therefore need assistance and respite care to alleviate these challenges. As rehabilitation services are limited in most African countries it is suggested that community-based and outreach services are provided in order to decrease the burden of care (Dambi & Jelsma 2014).

Participants reported experiencing social isolation and negative attitudes from their friends, family and the community. In most cases their friends and family members failed to accept the children with CP. The negative attitudes experienced were partly as a result of traditional and cultural beliefs and partners who, with or without their families, blaming the mothers for the condition of their children. This led to discrimination and prejudice against the mothers and their children (Sandy *et al*. 2013). Besides believing that disability has a spiritual cause (Wegner & Rhoda 2015), parents have also been accused of causing their children's disability (Ambikile & Outwater 2012). This response from society causes parents of these children to isolate themselves and keep their children at home. The mothers could have experienced less challenges had they been supported by their families (Adegoke *et al.* 2013).

As was, once again, found in the current study, attitudes towards children with disabilities were predominantly negative (Rosenweig & Huffstutter 2004). In most African cultures, pregnant women are often subjected to taboos and rituals to prevent them from having children with disabilities. A child born with any defect will be seen as a violation of such traditional belief systems, and the family will often be maltreated and looked down upon by the rest of the community members; in turn, the family ostracises, or discriminates against, the mother. Families believe if a mother gives birth to a child with disabilities, she should be blamed, because giving birth to a child with disabilities is seen as failure to follow traditional beliefs and cultures (United Nations Development Programme [UNDP] 1998). This finding could be linked to a misconception that disability occurs as a result of being associated with evil spirits and being punished by God or being involved in witchcraft Mothers of children with CP could therefore be ostracised as they were perceived to be doing some evil deed that needed punishment.

The physical environmental challenges experienced by nearly all the participating mothers were because of architectural and geographical features in the environment. Architectural features which restrict mobility for children with CP might be parts of buildings, landscaping, walkways, parking areas, including high curbs, lack of curb cuts or ramps, gravel walkways and narrow sidewalks (Urimubenshi & Rhoda 2011). These physical environmental challenges could result in problems accessing health- and other services (Komardjaja 2001). As the mothers often needed to carry their children this environmental barrier could further increase the physical stress they experience when caring for their children (Dambi & Jelsma 2014).

Some of the mothers in this study reported experiencing marital challenges owing to misunderstandings and conflict with spouses. The mothers reported that their husbands might leave them as soon as they discovered their children had CP. Others reported that they had constant fights with their husbands because of interference by relatives of their husbands, and in many cases they ended up divorced. Studies have shown that living with a child with disabilities can have profound effects on the entire family, and can affect all aspects of family functioning (Conman, Noonan & Reichman 2005; Swaminathan, Conman & Noonan 2005). A study in Canada that investigated marital relations amongst families of children with disabilities reported that relationships suffered unduly from the added stress of blame, guilt, and anxiety (Sobbey 2004).

Marital conflicts arose as partners blamed each other for the child's condition. Cultural beliefs in Zambia, just as in many African countries, dictate that physical disabilities of a child are caused by women (Skinner & Wesner 2007). As mentioned, society perceives that mothers are engaging in activities which were unacceptable and were therefore punished bearing a child with a disability. Marital problems could also be linked to the fact that the normal social lives of parents are affected when they have to care for a child with a disability resulting from neurological impairments (Lawal *et al*. 2014).

The study found that managing the chronic health problems of a child with CP along with coping with the demands of everyday life can have a detrimental effect on both the physical and psychological well-being of parents and caregivers.

Although the findings of the study are relevant it cannot be generalised to all mothers of children with CP in Zambia as the sample was selected from two specific rehabilitation centres. A community-based study might have revealed different findings.

## Conclusion

The mothers of children with CP experienced challenges which affected them physically as well as emotionally. They also experienced marital problems. As a result the mothers were isolated and lacked social support. It is therefore recommended that programmes and policies need to be implemented to support the mothers and inform society about various aspects of disability, especially in children. A family-centred approach to the management of children with CP should be taken, as this phenomenon impacts on the family as a whole. This will decrease the challenges experienced by mothers caring for children with CP.
